# PSYCHOSOCIAL FACTORS AND NONCOMMUNICABLE DISEASES IN OLDER SUB-SAHARA AFRICANS

**DOI:** 10.1093/geroni/igad104.0405

**Published:** 2023-12-21

**Authors:** James Muruthi, Margaret Adamek

## Abstract

Sub-Saharan Africa (SSA) has the fastest-growing proportion of older individuals globally. Concurrently, non-communicable diseases (NCDs) are a growing burden in the SSA aging population. An essential step towards reducing NCDs is an enhanced understanding of the various diseases and discussing their psychosocial risk factors. This session will provide a unique depiction of the occurrence and measurements of NCDs and their associations with psychosocial factors in aging SSA adults. Dr. Muruthi and colleagues present the psychometric properties of a Swahili-translated Kessler 6 scale in aging Kenyans. Drs Nwakasi and Esiaka discuss HIV health information acquisition among older Nigerian women who are cancer survivors. Dr. Esiaka and colleagues discuss psychosocial determinants of early cancer detection and survivorship among older Nigerian men. Drs. Adamek and Dr. Kotecho will present information about the extent of depression among older adults in Sub-Saharan Africa and an understanding of the factors contributing to high rates of geriatric depression. Findings from this session will help elucidate our understanding of NCDs and related psychosocial factors, a necessary step for developing robust evidence-based interventions to reduce non-communicable diseases (and their effects) among aging people in SSA.

